# The effect of environmental conditions on expression of *Bacteroides fragilis* and *Bacteroides thetaiotaomicron* C10 protease genes

**DOI:** 10.1186/1471-2180-12-190

**Published:** 2012-09-03

**Authors:** Roibeard F Thornton, Elizabeth C Murphy, Todd F Kagawa, Paul W O’Toole, Jakki C Cooney

**Affiliations:** 1 Department of Life Sciences, University of Limerick, Limerick, Ireland; 2 Materials and Surface Science Institute, University of Limerick, Limerick, Ireland; 3 Department of Microbiology, & Alimentary Pharmabiotic Centre, University College Cork, Cork, Ireland; 4 Present address: Department of Clinical Sciences, Lund University, Lund, Sweden

## Abstract

**Background:**

*Bacteroides fragilis* and *Bacteroides thetaiotaomicron* are members of the normal human intestinal microbiota. However, both organisms are capable of causing opportunistic infections, during which the environmental conditions to which the bacteria are exposed change dramatically. To further explore their potential for contributing to infection, we have characterized the expression in *B. thetaiotaomicron* of four homologues of the gene encoding the C10 cysteine protease SpeB, a potent extracellular virulence factor produced by *Streptococcus pyogenes*.

**Results:**

We identified a paralogous set of genes (*btp* genes) in the *B. thetaiotaomicron* genome, that were related to C10 protease genes we recently identified in *B. fragilis*. Similar to C10 proteases found in *B. fragilis,* three of the *B. thetaiotaomicron* homologues were transcriptionally coupled to genes encoding small proteins that are similar in structural architecture to Staphostatins, protease inhibitors associated with Staphopains in *Staphylococcus aureus*. The expression of genes for these C10 proteases in both *B. fragilis* and *B. thetaiotaomicron* was found to be regulated by environmental stimuli, in particular by exposure to oxygen, which may be important for their contribution to the development of opportunistic infections.

**Conclusions:**

Genes encoding C10 proteases are increasingly identified in operons which also contain genes encoding proteins homologous to protease inhibitors. The Bacteroides C10 protease gene expression levels are responsive to different environmental stimuli suggesting they may have distinct roles in the bacterial-host interaction.

## Background

The *Bacteroides* spp. are a group of Gram-negative anaerobes from the phylum *Bacteroidetes.* Members of the *Bacteroides* spp. occupy regions of the terminal ileum and colon, where they are a major component of the normal human gut microbiota. Although they are commensals, *Bacteroides* can cause opportunistic infections that may be triggered when the integrity of the mucosal wall of the intestine is compromised or breached, commonly leading to abdominal abscesses and bloodstream infections. Conditions that cause such a loss of intestinal barrier function include gastrointestinal surgery, perforated or gangrenous appendicitis, perforated ulcer, diverticulitis, and inflammatory bowel disease (IBD) [1]. Two of the most frequently isolated *Bacteroides* spp. from anaerobic infections are *B. fragilis* and *B. thetaiotaomicron.* Significantly, although *B. fragilis* accounts for only 4% to 13% of the normal human fecal microbiota it is isolated from 63% to 80% of *Bacteroides* infections. *B. thetaiotaomicron* on the other hand accounts for between 15% and 29% of the fecal microbiota but is linked with only 13% to 17% of infection cases [2]. This indicates that *B. fragilis* may be a more successful opportunistic pathogen then other related *Bacteroides* spp.

The majority of contemporary molecular studies on *Bacteroides* spp. focus on the mechanisms of polysaccharide utilization [2-4], with very few virulence mechanisms that contribute to the ability of *Bacteroides* spp. ability to act as opportunistic pathogens described. Among those that have, cell adherence, lipopolysaccharide production, and the production of neuraminidase, enterotoxin, and proteolytic enzymes have been proposed to play a role in *B. fragilis* pathogenicity [5]. *B. fragilis* also has the ability to produce several haemolysins [6]. Haemolysins have been identified as powerful virulence determinants in both Gram-positive and Gram-negative bacteria [7,8]. Recently we identified a large panel of orthologous genes encoding C10 proteases in the phylum Bacteroidetes, including a set of four paralogous genes (called Bfp1-4) in *B. fragilis*[9]. C10 proteases are papain-like cysteine proteases, and include Streptococcal pyrogenic exotoxin B (SpeB) from *Streptococcus pyogenes*, and Interpain A from *Prevotella intermedia*. Both of these enzymes have been implicated in virulence [10-13]. SpeB has been shown to cleave cytokines [14], activate the host matrix metalloprotease MMP-9, and to release kinin from kininogen [13]. In this way SpeB contributes to tissue damage and *Streptococcus pyogenes* invasion of the host [15]. Interpain A contributes to the pathogenesis of *P. intermedia* infections by inactivating the complement cascade through degradation of the complement factor C3 [12], a property also associated with SpeB [16]. Despite the obvious parallel functions of these orthologues, the activity of Btp proteases and their potential to contribute to virulence has yet to be determined.

SpeB and the Staphopains, papain-like proteases produced by *Staphylococcus aureus*, have been extensively studied with regard to regulation of gene expression, export and post-translational mechanisms [17-19]. These aspects of protease expression have yet to be investigated for papain-like cysteine proteases from members of the *Bacteroides* spp. The transcriptional coupling of the structural gene for the SpeB protease in *S. pyogenes* to a gene (*spi*) encoding a small specific inhibitor of SpeB [20], is remarkably similar to control of protease activity in some staphylococcal species [21]. The genes for the C47 type cysteine proteases Staphopain A and B, and their cognate inhibitors Staphostatin A and B, respectively, are contiguous and are co-transcribed [22]. Spi and the Staphostatins are thought to inhibit prematurely-activated proteases in the cytoplasm of their respective host cells, and thus prevent toxicity of the protease to the bacterial cell [20,23,24]. Despite the fact that SpeB and the Staphopains have a papain-like fold [10,25],[26], the inhibitors Spi and the Staphostatins are not related in sequence and have a different proposed mechanism of protease binding [20,21]. The SpeB-like proteases that we recently described in *B. fragilis* have Staphostatin-like inhibitors encoded either upstream or downstream of the protease gene, creating an unusual juxtaposition of C10 proteases and C47 protease type inhibitors. The *bfp* genes encoding the C10 proteases and the *bfi* genes encoding the inhibitors are co-transcriptionally coupled [9].

*B. fragilis* has been shown to differentially regulate virulence associated genes when occupying environmental niches other then the intestinal lumen. Among adaptive traits are aerotolerance and resistance to reactive oxygen species. These represent physiological adaptation of *B. fragilis* to its environment that may promote opportunistic infections by enhancing survival in areas outside the strictly anaerobic environment of the intestinal tract [27]. When *B. fragilis* was exposed to environmental oxygen, as might occur in the blood, a large number of genes for detoxification were induced such as catalase (*katB*) and superoxide dismutase (*sod*). Expression of these genes could prevent damage caused by reactive oxygen species [27]. The ferritin (*ftnA*) gene involved in iron acquisition was expressed at a low constitutive level when *B. fragilis* was grown under anaerobic conditions, but upon oxygen exposure, the *ftnA* message increased almost 10-fold in iron-replete medium [28]. This may be important for the ability of the organism to survive in an aerobic environment [28]. It has been proposed that the oxidative stress response regulator OxyR is required for full virulence in *B. fragilis*[27]. However, it is not known what factors are directly involved in this process. Although strictly anaerobic, *P. gingivalis*, which is phylogenetically close to *B. fragilis*, can also survive in the presence of atmospheric oxygen [29]. Significantly, two known virulence factors encoded by this organism, haemolysin (*hem*) and the cysteine protease gingipain A (*rgpA*), display elevated expression levels, 3.66-fold and 2-fold respectively, in the presence of atmospheric oxygen [30]. Thus it appears that cysteine protease gene expression in a related *BacteroidetesP. gingivalis* is sensitive to environmental cues including oxygen.

This study investigates how the expression of *B. fragilis* C10 protease genes responds to key changes in environmental stimuli, and thus indicates their potential involvement in pathogenesis and survival in the non-gut environs. In addition, expression analysis data is presented for a set of genes encoding newly identified and described C10 paralogues in *B. thetaiotaomicron*.

## Results

### Identification of a family of paralogous C10 protease genes in *B. thetaiotaomicron*

**Figure 1:**
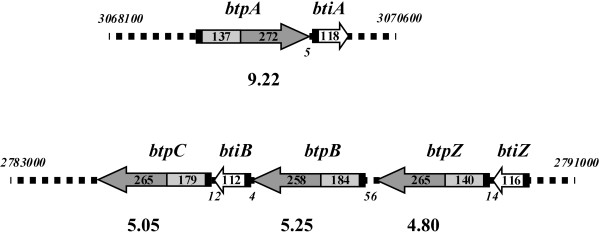
**Schematic diagram of two C10 protease loci in* B. thetaiotaomicron *VPI-5482.** The upper diagram represents the genomic region that includes *btpA*, the lower diagram the genomic region associated with the *btp* cluster. The proteases are represented by the larger open arrows. The propeptide region is represented by pale grey shading and the mature protease region by the darker grey. The white open arrows represent the stapostatin-like inhibitors. The black region at the 5’ end of each gene corresponds to the leader peptide encoding region of the gene. The co-ordinates for the region of the VPI-5482 are given by the numbers in italics above the DNA, the numbers in italics below the DNA are the intergenic distances. The numbers within the protease arrows are the numbers of amino acid residues in the propeptide and mature protease domain of the particular protein. The number below each of the proteases in larger font is the calculated pI of the respective mature protease.

**Figure 2:**
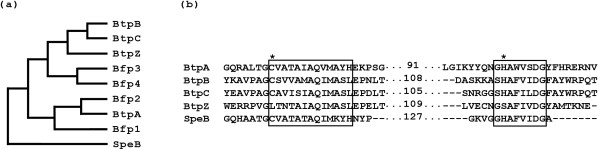
**Sequence relationship for C10 proteases from* B. fragilis*,* B. thetaiotaomicron *and* S. pyogenes*.** (**a**) Cladogram constructed for C10 protease sequences. (**b**) Amino acid sequence alignment of Btps from *B. thetaiotaomicron* with catalytic residues in SpeB.

By a combination of global homology-based approaches, supplemented by searching for active site motifs associated with cysteine protease activity, we identified 4 genes encoding homologues of the streptococcal C10 protease SpeB in the genome sequence of *B. thetaiotaomicron* strain VPI-5482. The genes were named *btpA* (BT2450), *btpB* (BT2219), *btpC* (BT2217) and *btpZ* (BT2220) for *Bacteroidesthetaiotaomicron*protease. Unlike *btpA*, the *btpBbtpC* and *btpZ* genes were found clustered together in the genome (Figure 1). The *btp* gene products ranged from 20.0% to 22.6% residue identity to SpeB, and 38.4% to 42.3% similarity (Table 1). The *btp* gene products were also found to share significant homology with the recently described [9] Bfp proteases of *B. fragilis* (18.3% to 27.6% identity and 38.4% to 49.8% similarity) (Table 1). Among the protein set, BtpA displayed the highest level of residue identity to Bfp1 and Bfp2, while BtpB, BtpC and BtpZ formed a separate cluster of related proteins (Figure 2(a)). Within this cluster, the most similar pair-wise alignment was between BtpB and BtpC, which were 54.3% identical and 2.5% similar (Figure 2(a) and Table 1). 

**Table 1:** **Identity and similarity matrix for*Bacteroides*C10 proteases**

	**SpeB**	**Bfp1**	**Bfp2**	**Bfp3**	**Bfp4**	**BtpA**	**BtpB**	**BtpC**	**BtpZ**
**SpeB**		**21.6**^a^	**18.0**	**22.6**	**23.3**	**22.6**	**20.0**	**21.6**	**21.1**
**Bfp1**	*43.0*^b^		**22.4**	**25.1**	**21.1**	**22.8**	**21.7**	**19.4**	**19.3**
**Bfp2**	*35.1*	*38.6*		**20.3**	**22.9**	**26.5**	**20.2**	**22.5**	**18.3**
**Bfp3**	*42.2*	*46.5*	*40.0*		**29.0**	**27.6**	**23.5**	**25.2**	**21.0**
**Bfp4**	*43.5*	*44.4*	*41.2*	*49.1*		**27.4**	**22.7**	**22.4**	**22.3**
**BtpA**	*42.3*	*45.8*	*44.1*	*49.8*	*49.3*		**22.4**	**21.9**	**22.8**
**BtpB**	*38.4*	*38.4*	*40.4*	*39.9*	*42.6*	*39.9*		**54.3**	**27.7**
**BtpC**	*38.4*	*39.0*	*41.9*	*43.6*	*44.5*	*40.6*	*72.5*		**29.2**
**BtpZ**	*41.3*	*40.6*	*41.2*	*41.5*	*45.4*	*42.3*	*47.3*	*47.7*	

^a^ Numbers in bold indicate percentage identity.

^b^ Numbers in italics indicate percentage similarity.

The four predicted proteases, BtpA, BtpB, BtpC and BtpZ, had deduced molecular mass values of 48714 Da, 52555 Da, 51669 Da and 47549 Da respectively, making them significantly larger molecules than SpeB (43174 Da). Similar to the Bfp enzymes, all four predicted proteases in *B. thetaiotaomicron* included an amino-terminal export signal with a cleavage site for signal peptidase II, suggesting they are lipoproteins. These leader peptides spanned residues 1–17 for BtpA, BtpB and BtpC and residues 1–19 for BtpZ. Proteases are typically expressed with a propeptide which promotes proper folding, and prevents inappropriate proteolytic activity. Guided by sequence comparisons with SpeB, all Btp proteins included primary sequence consistent with an N-terminal propeptide (residues 18–154, 18–201, 18–196 and 20–159 for BtpA, BtpB, BtpC and BtpZ respectively, and indicated in Figure 1). Also of note is the acidic nature of the clustered gene products BtpB, BtpC and BtpZ with pI values of 5.25, 5.05 and 4.80 for the predicted mature form of the proteins. This contrasts with the basic values of 9.22 for BtpA and 8.49 for SpeB. Sequence alignment and secondary structure predictions for the predicted proteins (BtpA, BtpB, BtpC and BtpZ) with the *B. fragilis* and *S. pyogenes* orthologues supports the idea that these proteases adopt a papain-like fold (data not shown). The catalytic Cys and His residues were conserved in BtpA, BtpB and BtpC (Figure 2(b)), though interestingly, the catalytic Cys residues was not preserved in BtpZ.

As noted above, co-expression with small, genetically linked protease inhibitors has emerged as a common theme for bacterial papain-like proteases [9,20,22]. Three candidate inhibitor genes were identified in open reading frames (ORFs) adjacent to the *btp* genes in *B. thetaiotaomicron* (Figure 1), with gene identification tags BT2452, BT2218, and BT2221. BT2452 was positioned 5 bp downstream of the *btpA* stop codon and named *btiA* (for *B.thetaiotaomicron* protease inhibitor A). Two *bti* genes, named *btiB* (BT2218) and *btiZ* (BT2221) were associated with the *btpBbtpC* and *btpZ* cluster of genes (Figure 1). A single copy of the *btiB* gene was interposed between *btpB* and *btpC*. *btiB* was located 4 bp downstream of the *btpB* stop codon, and the *bti* gene stop codon was 12 bp upstream of *btpC*. The stop codon of *btiZ*, the second *bti* gene in this cluster, was located 14 bp upstream of *btpZ*.

Sequence analysis of the predicted inhibitor proteins (BtiA, BtiB and BtiZ for the *btiA*, *btiB*, and *btiZ* genes respectively) indicated that all three proteins were likely to be exported through the inner membrane, and that the BtiA and BtiZ proteins were likely to be lipoproteins. Sequence comparison of the Bti proteins with the inhibitor-like sequences of *B. fragilis* 638R indicated 14.8% to 26.3% identity and 35.6% to 50.8% similarity (Table 2). Interestingly, BtiA and BtiB share the highest identity and similarity with Bfi1b (26.3% and 23.7% identity, and 48.9% and 50.8% similarity, respectively) (Table 2). In addition, the Bti proteins share common features with the Bfi proteins and the Staphostatins from staphylococci in that they are small, ranging from 116–138 amino acid residues, and would assume predominantly (predicted) β-sheet structures.

**Table 2:** **Identity and similarity matrix for*Bacteroides*inhibitors**

	**Spi**	**ScpB**	**SspC**	**Bfi1a**	**Bfi1b**	**Bfi4**	**BtiA**	**BtiB**	**BtiZ**
**Spi**		**16.4**^a^	**11.9**	**11.1**	**17.2**	**14.3**	**13.0**	**18.1**	**18.1**
**ScpB**	*41.7*^b^		**20.4**	**20.2**	**19.4**	**23.4**	**17.9**	**19.7**	**19.3**
**SspC**	*31.2*	*45.0*		**20.2**	**18.6**	**15.0**	**15.9**	**15.8**	**14.7**
**Bfi1a**	*26.7*	*38.8*	*45.7*		**20.3**	**20.4**	**20.1**	**14.9**	**18.8**
**Bfi1b**	*35.7*	*39.7*	*40.5*	*41.3*		**20.1**	**26.3**	**23.7**	**21.1**
**Bfi4**	*31.2*	*39.1*	*32.6*	*38.4*	*39.9*		**20.3**	**21.1**	**14.8**
**BtiA**	*29.0*	*35.9*	*32.8*	*40.5*	*48.9*	*46.4*		**21.7**	**17.1**
**BtiB**	*37.9*	*33.3*	*41.7*	*35.6*	*50.8*	*40.6*	*44.7*		**19.0**
**BtiZ**	*35.3*	*40.4*	*34.6*	*43.4*	*44.9*	*41.3*	*44.1*	*41.9*	

^a^ Numbers in bold indicate percentage identity.

^b^ Numbers in italics indicate percentage similarity.

Two of the C10 protease genes in *B. fragilis* were found on mobile genetic elements (MGE) [9]. However, extensive searches spanning 20 kb of the DNA either side of the *B. thetaiotaomicron* protease genes presented no convincing evidence for the presence of MGE-related genes in the vicinity of the Btp-Bti-encoding loci. However, this does not exclude the involvement of very large MGEs in the dissemination of these loci in *B. thetaiotaomicron.*

### The C10 proteases genes and predicted inhibitor genes in *B. thetaiotaomicron* are transcriptionally coupled

**Figure 3:**
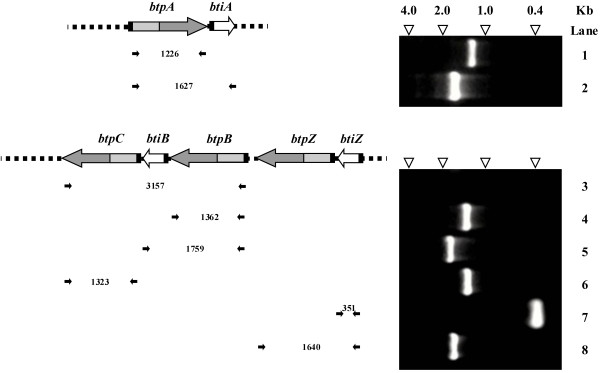
**Analysis of transcriptional coupling of C10 protease genes and inhibitor genes in* B. thetaiotaomicron *VPI-5482.** The left-hand side of the diagram shows the organization of the protease loci according to the colour scheme in Figure 1. The small black horizontal arrows represent the location of the PCR primer sites in the sequence, and the number between pairs of inverted arrows is the expected amplicon size in bp. The right-hand side of the diagram shows an agarose gel of the observed amplicons with the following lane assignments: Lane 1: *btpA*; Lane 2: *btpA-btiA*; Lane 3: *btpB-btpC*; Lane 4: *btpB*; Lane 5: *btpB-btiB*; Lane 6: *btpC*; Lane 7: *btiZ* and Lane 8: *btpZ-btiZ*. The top of the gel in on the right, with small white inverted triangles indicate the positions of the size markers in kb.

Analysis of mRNA isolated from *B. thetaiotaomicron* by Reverse-Transcriptase PCR showed expression of all four *btp* genes and the three *bti* genes. In addition, amplification of a 1.62 kb product demonstrated that *btpA* and *btiA* are co-transcribed as a single mRNA species (Figure 3, Lane 2). The analysis also demonstrated that *btpB* was transcriptionally coupled to *btiB* (Figure 3, Lane 5), supported by detection of a 1.76 kb amplicon and that *btpZ* and *btiZ* were transciptionally coupled (Figure 3, Lane 8), evidenced by a 1.64 kb amplicon. However, *btpC* could not be detected on a polycistronic mRNA with *btpB* and *btiB* (Figure 3, Lane 3), but appeared to be transcribed on a monocistronic message (Figure 3, Lane 6).

### The expression of *B. thetaiotaomicron* and *B. fragilis* C10 protease genes is responsive to changes in environmental conditions

**Figure 4:**
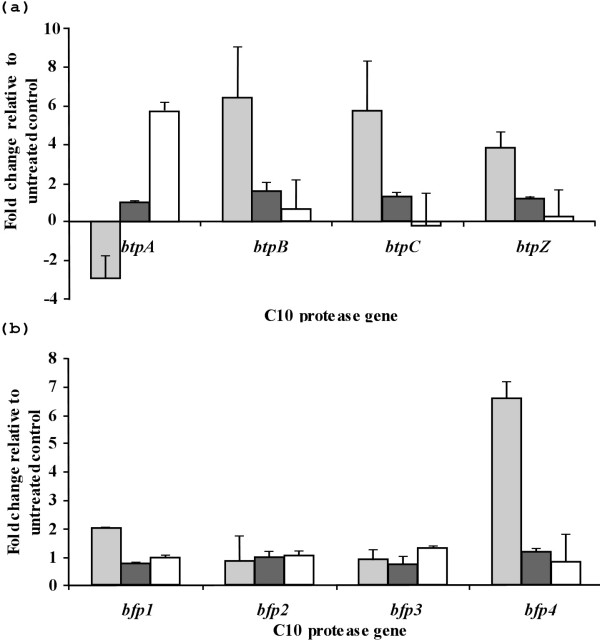
**Response of* B. thetaiotaomicron *and* B. fragilis *C10 protease genes to environmental stimuli.** The change in expression of the four *btp* genes in *B. thetaiotamicron* (**a**) and the four *bfp* genes in *B. fragilis* (**b**) was examined in response to atmospheric oxygen (light grey bar), bile (dark grey) and blood (white bar). In both plots, values between +/− 1 fold change indicate no significant alteration of gene expression compared to the control.

*B. thetaiotaomicron* was exposed to oxygen, or grown in the presence of either sheep blood or bile in order to mimic conditions the bacteria would encounter in the transition from the gut environment into the abdominal cavity. The change in the expression levels of the four C10 protease genes (*btpA, btpB, btpC* and *btpZ*) in response to these environmental stimuli was quantified by quantitative real-time PCR (qPCR). These data revealed a marked change in the expression levels of the four proteases genes under conditions of oxidative stress when compared to the control (Figure 4(a)). Expression of the *btpA* gene was inhibited upon exposure of the cells to oxygen, with the mRNA abundance being 3-fold lower than the control sample. The expression of the other protease genes however, was significantly up-regulated. The *btpB* gene expression level increased 6.4-fold, *btpC* increased 5.8-fold and *btpZ* increased 3.8-fold (Figure 4(a)), when compared to the control samples.

The expression of *btpA* was also observed to respond differently to exposure to sheep blood. Real time (qPCR) of mRNA/cDNA isolated from *B. thetaiotaomicron* cells grown on plates supplemented with 5% (v/v) sheep blood, indicated that *btpA* expression was significantly altered with a 5.6-fold increase in mRNA, while expression of *btpB*, *btpC*, and *btpZ* remained at constitutive levels (Figure 4(a)). Expression levels of all four *btp* genes were similarly non-responsive to bile exposure of the cells.

*B. fragilis* 638R was also exposed to atmospheric oxygen, or grown in the presence of sheep blood or bile, and the response in the expression levels of the *bfp* genes was measured. A qPCR analysis of *bfp* message indicated a marked shift in expression levels of *bfp1* and *bfp4* when exposed to atmospheric oxygen (Figure 4(b)). *bfp1* and *bfp4* mRNA production increased 2- and 6.6-fold respectively whereas, *bfp2* and *bfp3* mRNA expression remained unchanged from normal constitutive levels. No change in the expression levels of the four *B. fragilis bfp* genes could be detected when cells were grown in the presence of media supplement with blood, or with bile (Figure 4(b)).

### Exposure of *B. fragilis* to intestinal epithelial cells has no marked effect on C10 protease gene expression

*B. fragilis* have been shown to attach to gut epithelial cells [31]. To investigate whether the *B. fragilis bfp* genes respond to this attachment event, total RNA was isolated from *B. fragilis* after co-culturing with CaCO-2 cells, a human colonic epithelial cell line. Analysis of the bacterial mRNA for the levels of *bfp* message indicated that levels of *bfp* mRNA were unaffected after co-culturing with CaCO-2 cells (data not shown).

## Discussion

The *B. thetaiotaomicron* VPI-5482 genome was shown here to harbour genes for four members of the C10 family of papain-like cysteine proteases, three of which are genetically clustered, and associated with two staphostatin-like inhibitors. The fourth unlinked C10 protease gene was also associated with a staphostatin-like protein. Interestingly, the proteins encoded by the clustered genes were more closely related to each other than to BtpA, which had highest sequence identity to Bfp2, a protease in *B. fragilis*. Although no evidence was found to support the involvement of mobile genetic elements in the acquisition and evolution of these genes by *B. thetaiotaomicron*, it is nevertheless likely that the current genetic configuration has evolved by two separate horizontal gene transfer events. The first putative event was the acquisition of the *btpA* locus, and the second involved a single C10 gene insertion which is elsewhere in the genome. This was followed by subsequent gene duplication events yielding *btpB*, *btpC*, and *btpZ*, based on the fact that they share higher residue identity to each other than to *btpA*.

The *btpB* and *btpC* loci are the most closely related across the four paralogues encoding what are predicted to be functional proteases, with 54.3% and 72.5% overall amino acid sequence identity and similarity respectively (Table 1). The characteristic catalytic Cys residue of cysteine proteases is absent from BtpZ, indicating the *btpZ* gene product is not a functional protease, so the biological role of this molecule is unclear. Since all four *B. thetaiotaomicron* proteins include both a leader peptide and propeptide, it is likely that these proteins are exported across the inner membrane in a zymogen form, where the proteases would be expected to be anchored by virtue of predicted lipoprotein signal sequences and N-terminal residues. Based on the ‘+2 rule’ for lipoproteins, which relates the final location of a lipoprotein to the amino acid in the +2 position of the secreted protein [32], the likely cellular location of the Btp zymogens is coupled through a lipid moiety at the post-processing N-terminal Cys residue of the propeptide to the inner leaflet of the outer membrane. They would remain in this inactive form until an activation event occurred. As the proteases would thus have a periplasmic location, for them to contribute to virulence they must come into contact with the host. This could be achieved by a number of mechanisms (1) the presence of protease-specific transporters in the outer membrane, (2) by release of the proteases upon bacterial cell death and lysis, or (3) through vesicle-based transport, as previously described for *B. fragilis*[33]. In the case of the related organism *P. gingivalis* these vesicles have been associated with proteolytic activity [34,35]. It is therefore not unlikely that the proteases described in this paper could be exported by vesicles in a similar manner.

The Bti proteins also include predicted leader peptides, and BtiA and BtiB are likely to be lipoproteins, which would also most likely be associated with the outer membrane. BtiZ was not predicted to be a lipoprotein (the signal peptide for BtiZ has a signal peptidase I cleavage site) and it is therefore likely targeted to the periplasm of the Bacteroides cell. Having both membrane associated inhibitor and periplasmic inhibitors may be a strategy for maximizing protection afforded by these inhibitors against the C10 protease activity. Another possibility is that the BtiZ molecule is in the process of accumulating mutations and becoming non-functional in response to loss of BtpZ activity.

We have previously demonstrated the transcriptional coupling of *B. fragilis* C10 protease genes with those for staphostatin-like inhibitors [9]. In the current study transcriptional coupling was also identified for the *B. thetaiotaomicron btp* and *bti* genes by Reverse Transcriptase PCR. The *btpA* gene was found on the same message as *btiA*. Furthermore, transcriptional coupling was identified for *btpB* and *btiB*, and *btpZ* and *btiZ*. The *btpC* gene appears to be transcribed independently of adjacent *btp* and *bti* genes. Although, this study does not preclude that the *btpA, btpB and btpZ* genes could be transcribed independently of the *bti* genes, the data indicates a similar genetic linkage of these *btp* genes with staphostatin-like inhibitors as occurs in *B. fragilis*. As suggested for effective control of otherwise lethal proteases in streptococcus and staphylococcus, co-transcription with genes for cognate inhibitors ensures immediate availability and precise stoichiometry of the required inhibitor for the respective transcriptionally coupled protease.

*B*. *fragilis* and *B. thetaiotaomicron* are usually commensal components of the normal intestinal microbiota. However, *B. fragilis* cells adhered to epithelial cells in biopsy samples from IBD patients [36,37]. In addition, release of these organisms into other body sites can result in serious complications and they are associated with a range of extraintestinal infections [5]. Growth of *B. fragilis* in bile, blood and oxygen has previously been shown to enhance properties associated with increased virulence [6,27,38]. Bile is secreted into the small intestine as a normal part of fat digestion/metabolism. Previous studies on the exposure of *B. fragilis* to physiological concentrations of bile reported the increase of outer membrane vesicle formation and fimbria-like appendages, and increased expression of genes encoding antibiotic resistance-associated RND-type efflux pumps [38]. The same study showed that the bile salt-treated bacterial cells had increased resistance to a range of antimicrobial agents and as well as increased co-aggregation, biofilm formation, and adhesion to intestinal epithelial cells [38]. Bile is normally associated with small intestinal secretions. In the current study, *B. fragilis* and *B. thetaiotaomicron* were grown in the presence of physiological levels of bile (0.15% bile salts approximates to a concentration of 3.7 mM), reflecting concentrations found in the distal ileum (2 mM). These conditions did not alter the expression level of C10 protease genes in either organism. This suggests that in the large intestine, where the bile concentrations are considerably lower (0.09 to 0.9 mM), the production of these proteases is not likely to be responsive to residual levels of bile transiting from the small intestine.

The *oxyR* gene encodes a redox-sensitive transcriptional regulator of the oxidative stress response in *B. fragilis*[39]. It has been shown previously that *B. fragilis oxyR* mutants are attenuated in an intra-abdominal abscess infection model [27]. Thus the ability of *B. fragilis* to survive in oxygenated environments such as blood is thought to be linked with pathogenesis. Two of the *B. fragilis* C10 proteases (*bfp1* and *bfp4*) displayed increased expression levels when exposed to oxygen. The expression levels of the other protease genes (*bfp2* and *bfp3*) remained unchanged. Interestingly, genes encoding superoxide dismutase and an oxidoreductase can be found directly upstream of *bfp4*. These two genes encode proteins involved in the processing of reactive oxygen species and are also likely to be up-regulated in the presence of atmospheric oxygen. Three of the C10 protease genes in *B. thetaiotaomicron* were up-regulated significantly in the presence of oxygen, while *btpA* was down-regulated. These findings suggest that as the *Bacteroides* transit from the anaerobic environment of the gut lumen to a more aerobic environment, the bacterial cells respond and they alter the expression profile of these potential toxins. Oxidative stress is an obvious potential signal to the bacterial cell that it is leaving the anaerobic gut environment. Thus, it is possible that this cue triggers increased production of the C10 proteases as a means to combat the host immune system.

*B. fragilis* accounts for 55% of bacteraemia in adult patients resulting in systemic blood infections [40] and it is plausible that blood can act as an environmental signal for the expression of virulence factors in *Bacteroides* cells leaving the intestine. For example, stimulation of virulence gene expression by exposure to blood has been documented for *Streptococcus pyogenes*[41]. However, the study only sampled for a maximum of 3 hours growth in blood and did not detect an increase in expression of *speB*, the gene encoding the cysteine protease. SpeB is normally detected in culture supernatant in late-log phase growth. Other studies have suggested a role for SpeB in survival in blood [42]. Thus, the expression of C10 protease genes was also examined when *B. fragilis* and *B. thetaiotaomicron* were grown in the presence of blood. Only the expression of *btpA* from *B. thetaiotaomicron* increased upon exposure to blood, while the other *btp* genes were down-regulated. It was recently shown that the *Prevotella intermedia* Interpain A, a homologue of SpeB, and thus also of BtpA, has a role in the breakdown and release of haeme from haemoglobin [11]. Therefore, it is tempting to speculate that BtpA could carry out a similar function in iron acquisition.

The relatively late transition point in the qPCR for the proteases, combined with the observation that none of the protease genes tested showed differential expression upon exposure to CaCO-2 cells, makes it likely that in the environment of the gut these genes are transcribed at low levels. However, in situations where the bacteria are able to transit to the host tissue or blood stream these bacteria have the ability to produce select combinations of the C10 proteases in response to oxidative stress and the presence of blood, stimuli that would be encountered during transit. Interestingly, while *B. fragilis* produces four mature proteases that all have a basic (as distinct from acidic) character, the *B. thetaiotaomicron* proteases have distinct physicochemical properties. The predicted BtpA mature protease is basic in contrast to the predicted acidic character of BtpB, BtpC and BtpZ. This fact, and the mutually exclusive manner in which *btpA* and the clustered *btpB, btpC* and *btpZ* respond to the environment, suggests that these proteases may have very distinct targets and biological functions.

To date extensive attempts by us and others (J. Potempa, personal communication) to express these *Bacteroides* enzymes in a soluble and/or active format in *Escherichia coli* have been unsuccessful. There are a number of possible explanations, including the requirement for a chaperone for the correct folding of the proteases. Indeed, Nickerson and colleagues [43] suggest such a role for Staphostatin in the folding of Staphopains. In addition, activation of some bacterial proteases is not autoproteolytic but requires the action of additional proteases. This requirement has also been found in the staphylococcal system where the V8 serine protease is required for the maturation of the cysteine protease, Staphopain B, and in turn aureolysin is required to activate V8 protease [44]. Either of these scenarios would explain the difficulties in expressing active *Bacteroides* proteases in *E. coli*. Additional studies to overcome the issues experienced with recombinant protein expression are required, but although technically challenging, the characterization of these proteases at a biochemical level will improve the understanding of their function and potential roles in Bacteroides infections.

## Conclusions

The observation that bacterially encoded C10 (SpeB-like) proteases are more commonly co-transcribed with a potential inhibitor is thus established as a norm for cysteine protease systems in *Bacteroides spp.* The study has also established that these protease genes are expressed in two important members of the *Bacteroidetes* family, *B. fragilis* and *B. thetaiotaomicron*. The distinct expression patterns for each set of paralogs strongly suggest that proteases play diverse roles in the bacterial interaction with the host. In particular the response in gene expression to oxygen and blood exposure imply that the bacteria may alter the expression of these proteases as the bacteria transition from a commensal existence to that of an opportunistic pathogen.

## Methods

### Bacterial strains and culture conditions

*Bacteroides thetaiotaomicron* VPI-5482 was purchased from the United Kingdom National Culture Collection (UKNCC). *Bacteroides fragilis* 638R was a kind gift from Dr Sheila Patrick, Queen’s University, Belfast, Northern Ireland. Both *B. fragilis* and *B. thetaiotaomicron* were grown in an anaerobic chamber at 37 °C. Cultures were grown without shaking in Brain Heart Infusion (BHI) broth supplemented with 50 μg ml^-1^ hemin and 0.5 μg ml^-1^ menadione (BHI-HM). Media for plating was made from Brain Heart Infusion agar supplemented with 5% (v/v) defibrinated sheep blood.

For expression studies bacterial cells were grown for 20 hr in BHI-HM and subcultured into 30 ml BHI-HM media at a 1:20 dilution. Cells were grown for approximately 5 hr in an anaerobic gas jar at 37 °C until they reached mid-log phase. A BHI-HM subculture with no additional supplementation was used as a control. To test the bacterial response to atmospheric oxygen, mid-log phase cultures were incubated for an hour in a shaking aerobic incubator. In order to test the effect of blood or bile, cells from a 20 hr broth culture were spread plated onto BHI-HM agar plates supplemented with 5% (v/v) defibrinated sheep blood or 0.15% (w/v) porcine bile, respectively. Cells were also grown on unsupplemented BHI-HM agar as a control. The bacteria were grown for 24 hr under anaerobic conditions at 37 °C and then scraped from the plates using a plastic loop into 2 ml BHI-HM for RNA extraction.

### Bioinformatics and sequence analysis

Members of the C10 protease family from the *Bacteroides* spp. were detected by BLAST analysis [45]. Sequences were aligned using ClustalW [46] or T-Coffee [47]. Protein secondary structure was predicted using GorIV [48] and protein export signals were identified using LipoP [49]. Sequence relationships were analysed using MATGAT [50] and by construction of cladograms using DrawTree [51] with input information derived from dnd output files from T-Coffee.

### Total RNA isolation

RNA for quantitative Real Time PCR was extracted from *B. fragilis* 638R and *B. thetaiotaomicron* VPI-5482 cells using the hot phenol method [52]. Briefly, *Bacteroides* cells were grown in 50 ml of supplemented BHI medium to an OD_600_ of ~0.3. The cells were then harvested and resuspended in 1.5 ml of a solution containing 20 mM sodium acetate (pH 5.5), 0.5% (w/v) SDS, and 1 mM EDTA. After addition on to 1.5 ml of redistilled phenol (equilibrated with 200 mM sodium acetate, pH 5.5), the mixture was incubated at 68 °C for 5 minutes with gentle shaking. Following centrifugation at 10000 x g for 10 minutes the aqueous phase was re-extracted with 1.5 ml of phenol. The RNA was precipitated by adding 3 volumes of ethanol to the aqueous phase and chilled at −80 °C for 30 minutes. The RNA precipitate was collected by centrifugation at 10000 x g for 10 minutes and dissolved in 100 μl RNase free water. Further purification employed a column from an RNeasy mini Kit (QIAGEN, UK). Total RNA was subjected to DNase treatment using Turbo DNase (Ambion, UK). The RNA concentration was determined by measuring the optical density at 260 nm using a NanoDrop and the sample stored at −80 °C. The integrity of the RNA was confirmed by electrophoresis on a denaturing agarose gel or by using a Bioanalyzer (Agilent, USA).

### Reverse transcription analysis

Reverse transcription PCR (RT-PCR) for C10 proteases was performed using the Superscript III One-step RT-PCR system (Invitrogen, USA). Primers used in RT-PCR reactions are documented in Table 3. Primers were added to a final concentration of 200 nM and 200 ng of total RNA added. As a control for DNA contamination, RT-PCR reactions were set up where the control reaction only received primers after the reverse transcription step. Aliquots (5 μl) of all samples were analyzed by standard agarose gel electrophoresis.

**Table 3:** **Oligonucleotide primers used in the Reverse Transcriptase PCR study on*B. thetaiotaomicron*RNA**

**Primer**	**Sequence**
BtpA_F	CAGCAGGGATCCGATGACACAGAAGTAATGAAAC
BtpA_R	CAGCAGGAATTCTTATTTTATTATGTTAATATATGG
BtpB_F	CAGCAGGGATCCAATGATGAAGAAGGTTTGGATTTAC
BtpB_R	CAGCAGGAATTCCTTTTACAATATAATATCACAGATC
BtpC_F	CAGCAGGGATCCCTAGGTATGCAAGATAATCTG
BtpC_R	CAGCAGGAATTCTTATTTATTTATAATATTGTAAATC
BtpZ_F	CAGCAGGGATCCAAATATAATAGATGCAGAACGG
BtpZ_R	CAGCAGGAATTCTTATCTTTTTCTTATATCAGGTATAA
BtiA_R	CAGCAGGAATTCTTATTCTTTGGCCTTTTGTATTATAG
BtiB_F	CAGCAGGGATCCGAAGATGATGAAATATATATCAATG
BtiB_R	CAGCAGGAATTCTTATGGATTTTGCTTTATTGTATATG
BtiZ_F	CAGCAGCATATGAATTCTCCAAATTGTAATATAAAAA
BtiZ_R	CAGCAGGAATTCTTAAAGTTCAAAATCCCCCGATAAATC

### Quantitative real time PCR analysis

cDNA for quantitative Real Time PCR (qPCR) experiments were prepared using with 1–2 μg of RNA template and using the ImProm-II™ Reverse Transcription system (Promega, USA). For qPCR the cDNA template was used in a reaction mixture containing SYBR green with ROX as a reference dye (SYBR green 2x Master mix) (BioGene, UK) and gene-specific forward and reverse primers (Table 4). Reactions were performed using an ABI 7000 machine (Applied Biosystems, UK). qPCR amplification was performed using gene-specific primers with product sizes of approximately 150 bp. The reaction conditions for the qPCR were as follows: 95 °C for 10 minutes for the polymerase activation step, 40 cycles each of denaturing at 95 °C for 15 seconds, and annealing-extension at 60 °C for 15 seconds. To confirm primer specificity, melting curve analysis was performed with the following conditions; 95 °C for 15 seconds, 60 for 1 seconds, and 60 to 95 °C with a ramping rate of 0.5 °C per 10 seconds.

**Table 4:** **Oligonucleotide primers used in qRT-PCR with*B. fragilis*and*B. thetaiotaomicron***

**Primer**	**Sequence**
qBfp1_F	TTTAACAAGAAGCGGTGAACAAAGAA
qBfp1_R	TGCAATAGGAATACAACCCGCATAAT
qBfp2_F	CTACAAAGATAAAGCCACGGGAGCTA
qBfp2_R	TCTGTCTCCTCCCATAAAAACAGGTC
qBfp3_F	GAGGTTGTAAAAACGACACCAGCAAT
qBfp3_R	TGAGTATGCATAAATAGGTGCGGTTC
qBfp4_F	TCGTAGTGGGCAGTCAGGTTACTACA
qBfp4_R	ACTCTCCCAAACCATAGAATCCCAAT
q16S_Bf_F	GCGCACGGGTGAGTAACACGTAT
q16S_Bf_R	CGTTTACTGTGTGGACTACCAGG
qBtpA_F	CGTCTTCTACCCCTTGTTTGAGATGT
qBtpA_R	TTAAGTGACACGCTTCAATATCAGGAA
qBtpC_F	GTGCTGTTATTTCAATAGCACAGATT
qBtpC_R	TCTAGTTGTTTCAGAGGAAGGAGTTT
qBtpB_F	TGGTATAAAAATAGATTGGGAAGCAT
qBtpB_R	GGATGAGTACCAGAAAGGTCATAAAT
qBtpZ_F	AATTGTGGTAATATTCAAAAATGGAG
qBtpZ_R	AATATGCATTACTGCTAGAAGATTCG
q16S_Bt_F	TCACTGGACTGCAACTGACACTGAT
q16S_Bt_R	ACTCCCCAGGTGGAATACTTAATGCT

16S rRNA was amplified to serve as a comparator gene, against which expression of the genes of interest were normalized. Fold changes in gene expression were calculated by standard formula 2^(En-Et)-(Rn-Rt)^, where *En* is the cycle threshold (Ct) of the experimental gene (*e.g. bfp1*) in the control sample, *Rn* is the Ct of the reference gene (*i.e.* 16S rRNA) in the control sample, *Et* is the Ct of the experimental gene in the test sample and *Rt* is the Ct of the reference gene in the test sample [53]. qPCR was repeated on two different biological replicates and three technical replicates. Results were expressed as *n*-fold increase or decrease of expression upon exposure to different growth conditions, with a value of 1 representing no change in expression between the test and control samples.

### Growth of *B. fragilis* in the presence of CaCO-2 cells

Briefly, co-culturing experiments were performed as follows: CaCO-2 cells were maintained in Dulbecco modified Eagle's minimal essential media (DMEM, 25 mM glucose) supplemented with 10% (v/v) fetal calf serum, 1X amino acids (Gibco BRL, UK) 1% (w/v) L-glutamine and 10 μg ml^-1^ transferrin (CalBioChem, USA) and incubated in a humidified 37 °C incubator supplemented with 5% CO_2_. The cells were seeded at a density of 3 x 10^5^ cells ml^-1^ and allowed to grow to confluency for 4–7 days and then for a further 14 days by which time they become fully differentiated. *B. fragilis* was grown to mid-logarithmic phase as previously outlined. The cells (8 x 10^8^) were washed in PBS (140 mM NaCl, 2.7 mM KCl, 10 mM Na_2_HPO_4_, 1.8 mM KH_2_PO_4_) and resuspended in DMEM and finally placed in a T25 flask with CaCO-2 cells freshly rinsed in DMEM without antibiotics. These were incubated for 3 hours at 37 °C and 5% CO_2_. After co-culture, the *B. fragilis* cells were removed and the CaCO-2 cells were washed with DMEM to remove the non-adherent bacteria.

## Authors’ contributions

RFT and ECM performed and designed experiments, and interpreted data. TFK designed experiments and interpreted the data. PWOT designed experiments, analyzed data and co-wrote the manuscript. JCC conceived the study, designed the experiments, interpreted the data and co-wrote the manuscript. All authors read and approved the final manuscript.
